# Psychometric Properties of the Dutch Eyberg Child Behavior Inventory (ECBI) in a Community Sample and a Multi-Ethnic Clinical Sample

**DOI:** 10.1007/s10862-015-9482-1

**Published:** 2015-03-25

**Authors:** Mariëlle E. Abrahamse, Marianne Junger, Patty H. O. Leijten, Robert Lindeboom, Frits Boer, Ramón J. L. Lindauer

**Affiliations:** 1 De Bascule, Academic Center for Child and Adolescent Psychiatry, Amsterdam, The Netherlands; 2 Institute for Innovation and Governance Studies (IGS), School of Management & Governance, University of Twente, Enschede, The Netherlands; 3 Department of Child and Adolescent Psychiatry, Academic Medical Center, University of Amsterdam, Amsterdam, The Netherlands; 4 Department of Psychology, University of Utrecht, Utrecht, The Netherlands; 5 Child Development and Education, University of Amsterdam, Amsterdam, The Netherlands; 6 Division of Clinical Methods and Public Health, Academic Medical Center, University of Amsterdam, Amsterdam, The Netherlands

**Keywords:** Disruptive behavior problems, Eyberg Child Behavior Inventory (ECBI), Parent rating scale, Psychometric properties, One Parameter Logistic Model (OPLM), Rasch analysis

## Abstract

The Eyberg Child Behavior Inventory (ECBI) is an established parent rating scale to measure disruptive behavior problems in children aged between 2 and 16 years. The present study examined the psychometric properties of the Dutch translation, including analysis on the one-dimensional structure of the ECBI scales using item response theory. Data from two samples from the Netherlands were used, a community sample (*N* = 326; 51 % boys) and a multi-ethnic clinical sample (*N* = 197; 62 % boys). The one-dimensional structure of the ECBI Intensity and Problem Scales were confirmed in both of these samples. The results also indicated good internal consistency, test-retest reliability (community sample), and good convergent and divergent validity. The ECBI Intensity Scale was able to differentiate between diagnostic groups (no diagnosis, ADHD, ODD, and CD symptoms), demonstrating good discriminative validity. Findings support the use of the ECBI as a reliable measure for child disruptive behavior problems in a Dutch population. Suggestions for the optimal use of the both ECBI scales for research and screening purposes are made. Also, cultural issues regarding the use of the ECBI are discussed and additional research into the validity of the ECBI is recommended.

## Introduction

Persistently high levels of aggressive, oppositional, and impulsive behavior in young children are serious risk factors for negative developmental outcomes in adolescence and adulthood (Broidy et al. 2003; Burke et al. 2010). If left untreated, conduct-disordered behavior in young children can lead to serious difficulties in broad areas of functioning including difficulties in family, peer, school, and community interactions (Broidy et al. 2003). Long-term costs for education, mental health services, justice and social services are estimated at ten times higher for children with disruptive behavior disorders compared to children with no problems (Lee et al. 2012; Scott et al. 2001).

Early interventions are necessary to reduce the risk of serious disruptive behavior in adolescence and adulthood (Aos et al. 2004; Heckman 2006). Psychosocial interventions are considered the most effective treatment strategy for young children and their parents (Comer et al. 2013; Eyberg et al. 2008), however, to provide such treatment, adequate early screening of behavioral problems in children is necessary. Parent rating scales are the most efficient and commonly used method for screening behavior problems in young children (Funderburk et al. 2003).

### Eyberg Child Behavior Inventory (ECBI)

The ECBI (Eyberg and Pincus 1999) is widely used for early screening of disruptive child behavior within both clinical and research settings. The ECBI is a parent rating scale, designed to measure the level of disruptive behavior in children aged between 2 and 16. The ECBI has several strengths. Firstly, the ECBI has been shown to be sensitive in measuring the effect of treatment on disruptive behavior problems (Eisenstadt et al. 1993; Nixon et al. 2004). Secondly, the ECBI is short (36 items) and easy to complete. It contains short and concisely described child behaviors with little room for interpretation, which makes it easy to understand. Contrary to more comprehensive instruments like the 100-item Child Behavior Checklist (CBCL; Achenbach and Rescorla 2000), the ECBI requires less concentration to complete. Therefore, the ECBI is particularly suited for screening in lower educated families. Moreover, the ECBI is unique in its use of two different scales to assess disruptive child behavior: the Intensity Scale (IS) and the Problem Scale (PS). For each item, parents are asked how often their child displays this behavior (IS) and whether or not they find this behavior problematic (PS).

The ECBI has been translated into several languages and is used across the United States (US) and Europe. The ECBI is also used in Japan, South Korea and China. The reliability and validity of the ECBI is supported in over 20 studies across cultures and countries (e.g. Funderburk et al. 2003; Sivan et al. 2008). High internal consistency of the two scales (alphas > .90), has been demonstrated in several socio-demographic subgroups (Colvin et al. 1999). There is evidence suggesting the ECBI has good retest reliability (*r* = .75) over a 10 month period (Funderburk et al. 2003). Normative data from community samples are available (Colvin et al. 1999) and indicate that mean ECBI scores are considerably lower in Northern European countries, including Sweden (ECBI IS mean = 88.2; Axberg et al. 2008) and Norway (ECBI IS mean = 89.9; Reedtz et al. 2008), compared to the US (ECBI IS mean = 96.6; Colvin et al. 1999).

There is also evidence that the ECBI Intensity Scale correlates strongly with other well-known questionnaires assessing child behavior problems, such as the CBCL (Achenbach and Rescorla 2000) and the Strengths and Difficulties Questionnaire (SDQ; Goodman 1997), suggesting good construct validity. In a non-clinical Swedish sample of children between 3 and 10 years of age correlations between the ECBI Intensity Scale and the total difficulties scale of the SDQ were 0.68 (Axberg et al. 2008). In a clinically referred US sample of children between 4 and 16 year of age correlations between the ECBI Intensity Scale and the CBCL Externalizing Behavior scale were 0.75 (Boggs et al. 1990). In line with the expectations, correlations with scales measuring internalizing behavior problems were lower than correlations with scales measuring externalizing behavior problems (Axberg et al. 2008; Butler 2011). With regards to the discriminative validity of the ECBI, in the clinically referred US sample as described by Weis and colleagues (2005), the Intensity Scale distinguished between groups of children with no significant externalizing problems, children with inattentive and oppositional behavior symptoms, and children with more serious behavioral problems.

Although the ECBI is widely used, and the evidence for validity across countries is strong, no evidence regarding the psychometric properties of the ECBI is available in the Netherlands and most other European countries. Adequate use of the ECBI for screening and treatment evaluation purposes requires knowledge regarding its psychometric properties in a Dutch community and clinical population. The goal of the present study was to examine the psychometric qualities of the ECBI scales in terms of internal consistency, test-retest reliability, reproducibility, convergent, divergent, and discriminative validity. We investigated these psychometric properties in two samples: a community sample and a clinical sample. Considering the international evidence suggesting that the Intensity and Problem Scales of the ECBI have good psychometric properties, we hypothesized that we would find similar results.

### Dimensionality of the ECBI

The ECBI is a screening tool with established cut-offs (Eyberg and Pincus 1999) and is primarily designed to assess a single dimension of disruptive behavior problems (Colvin et al. 1999; Eyberg and Robinson 1983). However, the ECBI contains items that reflect the symptoms of Attention Deficit Hyperactivity Disorder (ADHD), Oppositional Defiant Disorder (ODD), and Conduct Disorder (CD) as described by DSM-5 (American Psychiatric Association 2013). Evidence regarding the factor structure of the ECBI Intensity Scale is inconsistent. Burns and Patterson (1991, 2000) identified three clinical meaningful dimensions of the ECBI within a community and clinically referred US sample: Inattentive Behavior, Oppositional Defiant Behavior Toward Adults, and Conduct Problem Behavior. These findings suggest that the ECBI can be used to differentiate between behavior disorders within the externalizing behavior spectrum (Weis et al. 2005). This three-factor structure was replicated in several studies including community and clinical samples, and demonstrated both predictive and discriminant validity (Axberg et al. 2008; Weis et al. 2005).

Other researchers, however, failed to replicate these results. Gross et al. (2007) found more support for the validity of the ECBI as a one-dimensional measure for child behavioral problems. More recently, in a community sample, including low income families from different cultural backgrounds and of different ethnicities Butler (2011) failed to replicate the results for a three-factor structure of the ECBI and suggested that these factors are not used for screening and treatment outcome research.

Previous studies exploring the factor structure of the ECBI used factor analysis. However, factor analysis is correlation-based and strongly dependent on the study sample used. Results may therefore vary from sample to sample. Currently, the three-factor structure of the ECBI is not used in treatment outcome research, and there is still a preference for using the ECBI as a one-dimensional scale for measuring child disruptive behavior (Comer et al. 2013; Michelson et al. 2013). Additional research on a larger sample of children is however needed to shed light on the preferred unidimensional use of the ECBI Intensity and Problem Scales. The use of a larger sample would provide the opportunity to apply modern methods of scale validation, such as Rasch analysis or Item response theory (IRT) analysis, which produce results that are less sample-dependent.

In summary, the other goal of the study was to test the one-dimensional structure of the ECBI scales using modern test analysis techniques to provide more information on the dimensional structure of the ECBI.

## Method

### Participants and Procedure

Two samples were included in the present study, a community sample (*n* = 326) and a clinically referred sample (*n* = 197). Informed consent was obtained from all individual participants included in the study.

#### Community Sample

To assess behavior problems in a community sample, parents were recruited at child day care centers, primary schools and through social networks in several regions of the Netherlands. Teachers or day care workers provided parents with the ECBI and an additional demographic questionnaire was used to obtain background information about the informants and the children in the study. In this sample undergraduate students distributed 555 questionnaires and 183 questionnaires were returned, indicating a response rate of 33 %. This low response rate could be a consequence of different levels of motivation from teachers. The remaining 143 questionnaires were retrieved following digital distribution, as some schools sent parents an e-mail including a link to complete the questionnaires online. For this sample, however, no response rate was available, because the total number of parents receiving this e-mail was unknown. To assess the test-retest reliability of the ECBI, participating parents were contacted by e-mail to fill out the ECBI again 6 months later. To motivate the parents to participate for a second time, a gift card was provided as a raffle prize. The response rate for this 6-month follow-up was 50.6 %.

Attrition analyses on the non-responders from the assessment of test-retest reliability indicated that parents of children with a non-western background were less likely to respond at the 6-month follow-up (*χ* ^2^ (1) = 9.19, *p* < .01). However, no differences between responders and non-responders were found on other demographic characteristics (child age, child gender, rater’s gender and education). The baseline ECBI scores on the Intensity and Problem Scales also did not differ significantly (IS; *t* (324) = 1.76, *p* = .08, PS; *t* (324) = 1.76, *p* = .08) between responders and non-responders.

In total, 326 parents (86.8 % mothers) of 2 to 8-year old (*M* = 5.54, *SD* = 1.40) children completed the ECBI. The sample included 165 boys and 161 girls. The classification criteria of Statistics Netherlands (2013) were used to classify each child’s ethnic background resulting in three categories. Most of the children (90.8 %) were classified as Dutch, 4.9 % were classified as other western (for example Spanish or French), and 4.3 % was classified as non-western. Parental education was categorized as low (no education or primary education), middle (secondary education) or high (higher academic education) (Statistics Netherlands 2014).

#### Clinical Sample

Families were referred or recruited to take part in a parent management training intervention which aimed to help with their child’s disruptive behavior problems and were involved in two treatment evaluation studies. Most families (*n* = 111) were referred to mental health services by a general practitioner or a child welfare organization. The other families (*n* = 96) were recruited following an information meeting at their child’s school. Families who perceived problems in parenting were asked to participate in the treatment evaluation study. Due to the fact that participation in this group was voluntary, no refusal rates are available. In the referred group, sixteen families (14 %) refused to participate in the study, however, no demographic information is available for this group. A medical ethics committee approved these studies. All participants (*n* = 197) lived in an urban region in the Netherlands. All parents who participated provided informed consent and were contacted to complete a demographic questionnaire, the ECBI, and the SDQ in one sitting prior to beginning treatment. Participants received a small amount of compensation (€10 or €15 gift card) for their participation. Most parents received and returned the questionnaires by post, but some parents completed the questionnaires during a home visit by the researcher.

The overall sample consisted of 277 parents and 197 children (122 boys and 75 girls) aged between 2.5 and 8.5 years (*M* = 5.53, *SD* = 1.36). The dates of birth of four children were unknown. For these children we were therefore not able to calculate their exact age. For all children (*N* = 197) the mother was involved in the study. Additionally, for 79 children (40.1 %) both parents completed the questionnaires, because the father was also involved in treatment. The sample consisted of participants from a range of ethnic backgrounds, 54.7 % of the children were classified as Dutch, 1.8 % was classified as other western and 43.5 % was classified as non-western (mainly Moroccan and Turkish families).

### Measures

#### Eyberg Child Behavior Inventory (ECBI: Eyberg and Pincus 1999)

The Intensity Scale (IS) and the Problem Scale (PS) of the ECBI were included in this study The Intensity Scale measures the frequency of child behavior problems using a 7-point Likert scale (1 = *never* to 7 = *always*) and the overall score reflects the severity of disruptive behavior. The Problem Scale measures parental tolerance for their child’s misbehavior, which is measured by asking parents whether or not they view each of the described behaviors as problematic, using a dichotomous scale (1 = *yes*, 0 = *no*). The Dutch ECBI was translated and back-translated which resulted in a final version being approved by Psychological Assessment Resources (PAR). In the clinical sample, participant level data from the two treatment evaluation studies were pooled and two slightly different versions of the Dutch ECBI translations were used (i.e., minor differences in the wording of 12 of the 36 items). For example, item 11 (*Argues/Discuses with parents about rules*). Considering that differences were minor and preliminary analyses revealed no impact, we can assume that there were no effects of combining these two versions for the current study.

#### Strengths and Difficulties Questionnaire (SDQ)

All parents in the clinically referred sample filled out the SDQ, a brief 25-item questionnaire which assesses emotional and behavior problems in children from 3 to 16 years of age (Goodman 1997). The SDQ contains three response categories (0 = *not true*, 1 = *somewhat true* and 2 = *certainly true*) and has a Total Difficulties scale. The SDQ consists of five subscales all containing the sum of five items. In the current study the internal consistencies (Cronbach’s alpha’s) for all SDQ scales when completed by mothers were *α* = .66 (Emotional Symptoms), *α* = .57 (Conduct Problems), *α* = .79 (Hyperactivity/Inattention Problems) *α* = .34 (Peer Problems) and *α* = .73 (Prosocial Behavior). The internal consistencies for the scales when completed by fathers were comparable and ranged between *α* = .37 (Peer Problems) and *α* = .78 (Hyperactivity/Inattention Problems). The SDQ scale for conduct problems (SDQ-CON) and the scale for hyperactivity and impulsiveness (SDQ-HYP) were converted to a pooled scale (SDQ-CON+HYP), as was done by Axberg and colleagues (2008). This allowed for a comparison of the ECBI items, which were included in both scales.

#### Symptoms for Clinical Diagnosis

For most children in the clinically referred sample (*n* = 137) a diagnostic assessment was conducted as part of the baseline assessment for the treatment evaluation study. For some families no diagnostic information was collected due to differences in clinical practice or practical issues, for example some families were not reached for the diagnostic interview before the start of treatment. Children were assessed for the presence of attention or hyperactivity problems, oppositional defiant behavior and conduct problem behavior based on the diagnostic criteria of the fourth edition of the Diagnostic and Statistical Manual of Mental Disorders (DSM-IV; American Psychiatric Association 1994). Trained clinicians and psychiatrists administered these interviews and observations.

### Statistical Procedure

All analyses were performed in SPSS version 19 or 21. Parents who did not complete all of the ECBI items (missing ≥4 items per scale) were excluded from the study, as is advised in the professional manual by Eyberg and Pincus (1999). In total, 7 children were excluded from the community sample and 28 children were excluded form the clinical sample. Chi-square tests revealed no differences in demographic characteristics between participants who had incomplete questionnaires and those with less than 4 missing items or no missing items. Also, as described in the manual guidelines, missing values were replaced with *1 (Never)* for the Intensity Scale and *0 (No)* for the Problem Scale (Axberg et al. 2008; Eyberg and Pincus 1999). The most common missing items were item 25 and item 27 (*Verbally / psychically fights with sisters and brothers*), because these questions were not applicable for parents with just one child.

In the community sample, 25 families had one or two missing items which were replaced, and in the clinical sample 24 families had one, two or three missing items which were replaced. Preliminary analyses with the participants who had complete ECBI’s revealed no influence of the item replacement on the internal consistency and mean ECBI scores. Chi-square tests and one-way ANOVAs also revealed no significant differences in the demographic characteristics of the parents and children who had complete questionnaires and those who did not.

Statistical analyses were performed is three stages. First, the unidimensional structure of the ECBI scales was tested in order to allow for exploration of the other psychometric properties of the ECBI in the appropriate scales. The dimensionality of the ECBI scales was examined using item statistics, including item-total correlations and internal consistency (Cronbach’s alphas). An exploratory factor analysis (EFA) was conducted as a preliminary analysis in order to examine the dimensional structure of the ECBI scales. Factors were extracted via principal axis factoring with oblique rotation. Oblique rotation was chosen, because it was expected that the factors measuring externalizing behavior would be correlated (Nolan et al. 2001). The EFA was run without specifying the number of factors. Factor loadings, scree plots and eigenvalues using the Kaiser-Guttman rule (Fabrigar et al. 1999) were examined and a parallel analysis (Horn 1965) was conducted to determine if the ECBI contained a dominant first factor.

Subsequently, item response theory methods, a specific extension of the Rasch measurement model (Verhelst and Glas 1995; Verhelst et al. 2005) were used to confirm the one-dimensional structure of the ECBI Intensity and Problem Scales. This method requires a large number of observations (preferably >300). Therefore, the community and clinical sample were combined for these analyses. The item scores on the community sample also showed too limited variation to perform a meaningful IRT analysis with this sample alone. Contrary to the basic Rasch model (1960) that assumes equal discriminative capacities for each test item, the extension of this model, the one-parameter logistic model (OPLM), allows individual items to vary by assigning item weights according to their capacity to discriminate between individuals on their level of problem behavior. Weights may vary between 1 (low discriminative capacity of an item) to 5 (very high discriminative capacity of an item). Like the basic Rasch model, OPLM requires the answer categories of the scales to have a dichotomous structure. Dichotomization was appropriate for this data, because a rating scale analysis showed disordered rating scale categories. For example, higher item categories showed lower item threshold difficulties than lower adjacent categories for many items. Hence, ECBI Intensity Scale items were first dichotomized into two categories indicating a low and high frequency of a specific problem behavior. In order to have an adequate distribution between categories and based on the distribution of the data, it was chosen to classify an item score of 1, 2, and 3 as 0 *(low)* and an item score of 4, 5, 6, and 7 as 1 *(high)*. Conditional maximum likelihood estimation methods were used to estimate the item and person parameters for the ECBI scales. Item fit to the OPLM model (after testing fit to the basic Rasch model) was tested using item-oriented fit statistics (S tests) that examine observed and expected numbers with a given item score conditional on the problem behavior level as measured with the ECBI. Overall goodness of fit of all item responses to the one-dimensional model was tested with the R1c statistic, a chi-square based test using *p* > .05 as a criterion for model fit, meaning that the observed item responses do not differ significantly from the expected item responses in the unidimensional model.

After testing for the one-dimensional structure, additional psychometric properties were examined in both the community and clinical samples. These analyses included correlations, and the calculation of the ECBI Intensity and Problem Scale means for the total samples and subgroups. Differences between groups were examined using *t-*tests and one-way ANOVAs. The reproducibility of the ECBI items score from the test-retest reliability assessment was evaluated using quadratic weighted kappa coefficients for the ordinal structure of the ECBI Intensity Scale and unweighted kappa coefficients for the dichotomous structure of the ECBI Problem Scale. Additionally, the reproducibility of the ECBI sum scores (total Intensity Scale and Problem Scale) was evaluated using intraclass correlations, using a two-way mixed model (Fleiss and Cohen 1973).

Finally, the discriminative validity was evaluated in the clinical sample to test the ability of the ECBI Intensity and Problem Scales to discriminate between significant DSM-IV symptoms with regards to ADHD, ODD, and CD. One-way ANOVAs were used to evaluate differences in mean scores between these diagnostic groups.

## Results

### Dimensionality of the ECBI Scales

The internal consistency (Cronbach’s alpha’s) of the ECBI scales was high in both the community sample (COS; IS & PS; *α* = .93) and the clinical sample (CLS; IS; *α* = .93, PS; *α* = .91). Also, coefficients of the father reports in the clinical sample were almost equal (IS; *α* = .93, PS; *α* = .92). The corrected item-total correlations indicated similar results in both samples, with coefficients for the ECBI Intensity and Problem Scales ranging from 0.09 (item 36, *Wets the bed*) to 0.73 (item 9, *Refuses to obey until threatened with punishment*). The median of these scores ranged from 0.46 (CLS-PS) to 0.55 (CLS-IS), indicating an overall satisfactory item-total correlation.

Subsequently, the EFA on the ECBI Intensity Scale revealed a dominant first factor, which explained 30.7 % of the variance in the community sample and 32.1 % of the variance in the clinical sample. The eigenvalue analysis identified nine factors in both samples with eigenvalues >1. The percentage of explained variance for the eight additional factors ranged from 2.8 to 7.4. A parallel analysis extracted ten factors in the community sample and six factors in the clinical sample. In both samples, however, a dominant first factor was identified based on the raw data eigenvalues (for example 11.2 for the first factor compared to 2.1 for the second factor in the clinical sample). The EFA of the ECBI Problem Scales revealed similar results. For this scale a dominant first factor was also found explaining 30.0 % of the variance in the community sample and 25.3 % of the variance in the clinical sample. Eleven factors with eigenvalues ≥1 were identified in the community sample compared to 10 for the clinical sample. Again for this ECBI Problem Scale these additional factors had low percentages of unique explained variance ranging from 2.8 to 7.6. The parallel analysis also revealed a high number of factors for both community (19) and clinical samples (9), however, based on the raw data eigenvalues for the ECBI Problem Scale a dominant first factor was again identified.

**Fig. 1:**
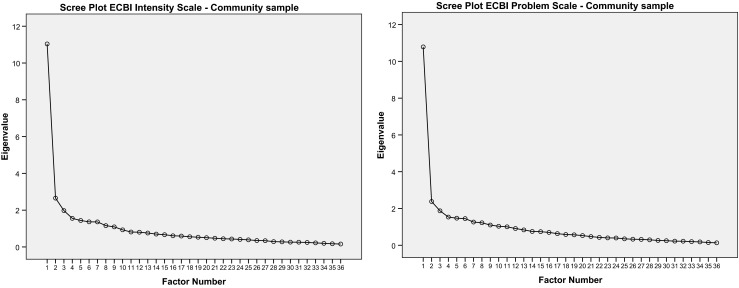
Scree plots ECBI Intensity and Problem Scale for community sample

**Fig. 2:**
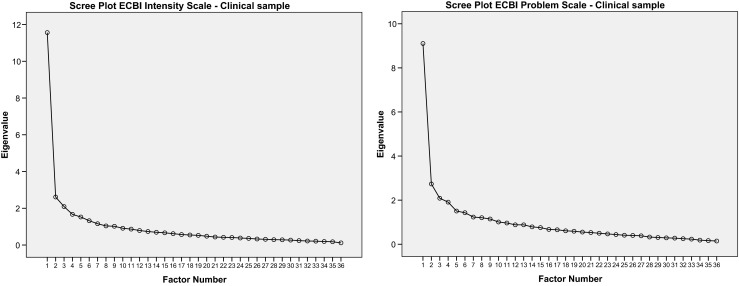
Scree plots ECBI Intensity and Problem Scale for clinical sample

In general, factor loadings of the ECBI Intensity and Problem Scale items on the first dominant factor were satisfactory and ranged from 0.09 (item 36, *Wets the bed*) to 0.76 (item 10, *Acts defiant when told to do something*). The median factor loading scores ranged from 0.50 (CLS-PS) to 0.59 (CLS-IS). In both samples ECBI Intensity and Problem Scales factor loadings for item 36 (*Wets the bed*) were low (<.25). Item 21 *(Steals)* had poor factor loadings (<.30) on the ECBI Intensity Scale. Figures 1 and 2 present the scree plots for the ECBI scales which also confirm the presence of one dominant factor. Therefore, we used the Rasch model to further investigate the one-factor structure of the ECBI Intensity and Problem Scales.

The community and clinically referred sample data were combined to conduct the Rasch analysis resulting in a total sample size of *N* = 514 for the ECBI Intensity Scale and *N* = 481 for the ECBI Problem Scale. The initial Rasch analysis revealed insufficient item fit of the ECBI scales to the model. Additionally, the extension of the Rasch model (OPLM) was conducted, allowing the items to differ in their discriminative capacity. Items were weighted for their ability to discriminate between individual participants on their level of problem behavior on the ECBI scales. After weighting the items, there was good overall fit on the OPLM for both ECBI scales. The observed and expected scores using the model were similar. The R1c goodness of fit statistic for ECBI Intensity Scale was χ^2^ (105) = 115.1, *p* = .237. For the ECBI Problem Scale the R1c statistic was χ^2^ (105) = 83.6, *p* = .936. These results indicate that the 36 items of the ECBI Intensity and Problem Scale constitute one dimension. Using the OPLM, items can be weighted for their impact. Table 1 presents the weights for the specific items of the ECBI scales. For the ECBI Intensity Scale item 13 (*Has temper tantrums*) and item 19 (*Destroys toys and other objects*) were classified with the highest weights (5). This indicates that when a parent scores 4, 5, 6 or 7 (after dichotomization 1) on these specific items, a higher total score of problem behavior is expected. For the ECBI Problem Scale items 8 (*Does not obey house rules on own*), 10 (*Acts defiant when told to do something*), and 11 (*Argues with parents about rules*) had the highest weights.

**Table 1:** Classification of proposed weighted scores per item for the ECBI Intensity and Problem Scale based on the extended Rasch model (OPLM) outcomes

Weights	Intensity scale item	Problem scale item
1.	2, 36	2, 36
2.	1, 4, 6, 7, 16, 21, 25, 26, 27	1, 4, 16, 22, 33
3.	3, 5, 12, 15, 18, 20, 22, 23, 24, 32, 33	3, 5, 6, 7, 15, 18, 20, 21, 23, 24, 25, 27, 28, 29, 30, 32, 34, 35
4.	8, 9, 10, 11, 14, 17, 28, 29, 30, 31, 34, 35	9, 12, 13, 14, 17, 19, 26, 31
5.	13, 19	8, 10, 11

After dichotomization of the ECBI Intensity Scale into 0 and 1 and using these weights a maximum of 111 can be scored. For the ECBI Problem Scale a maximum of 113 can be scored

### Psychometric Properties

#### Descriptive Statistics

In both the community and clinical samples the correlations between the ECBI Intensity and Problem Scale were significant (COS; (*r* (304) = .62, *p* < .001), CLS mother reports; (*r* (175) = .75, *p* < .001), CLS father reports (*r* (73) = .67, *p* < .001). Respectively, they shared 38, 45 and 56 % of the variance, indicating a moderately strong correlation. In the community sample, standardized positive values for skewness and kurtosis were significant on both the ECBI Intensity and Problem Scales, indicating a non-normal distribution of the scales. For the clinical sample, for mother and father reports, these values revealed a normal distribution.

Table 2 shows the mean scores for the ECBI Intensity and Problem Scales for both samples, organized by children’s age, sex and ethnicity, and informant’s gender and educational level. Subgroup analyses revealed significant sex differences in the ECBI Intensity Scale in the community sample; boys had higher scores than girls (*t* (324) = 2.32, *p* = .02) and in the ECBI Problem Scale in the Clinical Sample (*t* (175) = 2.50, *p* = .01). The effect sizes for these differences were small (COS; *d* = .26, CLS; *d* = .38). Additionally, in the clinical sample one-way ANOVAs revealed a significant effect for child ethnicity on the mother ECBI Intensity Scale (*F* = (2165) 10.88, *p* < .001). Mothers of children with a Dutch background reported a higher frequency of behavior problems than mothers of children of a non-western background.

**Table 2:** Mean and standard deviations of ECBI Intensity and Problem Scale scores for the community sample and clinical sample organized by subgroups

	Community sample (*N* = 326)				Clinical sample (*N* = 197)			
	Intensity score		Problem score		Intensity score		Problem score	
	*n*	*M (SD)*	*n*	*M (SD)*	*n*	*M (SD)*	*n*	*M (SD)*
Child age								
2–5	149	86.1 (24.4)	141	4.0 (6.0)	118	129.9 (34.5)	110	15.3 (8.5)
6–8	177	84.3 (23.5)	163	4.2 (6.1)	72	130.2 (34.7)	64	15.6 (9.0)
Child sex								
Girl	161	82.0 (23.7)^a^	151	3.9 (6.0)	74	124.2 (33.8)	70	13.5 (8.4)
Boy	165	88.1 (23.7)^a^	153	4.4 (6.1)	119	133.6 (34.2)	107	16.7 (8.6)
Child ethnicity								
Dutch background	296	85.5 (24.4)	277	4.1 (6.1)	92	142.1 (31.1)^b^	83	16.9 (7.5)
Western background	16	84.9 (19.1)	16	4.6 (4.7)	3	144.0 (24.6)	3	21.3 (4.2)
Non-western background	14	76.6 (15.9)	11	5.6 (7.5)	73	119.3 (32.0)^b^	66	14.7 (9.3)
Informant								
Mother	283	85.3 (24.5)	263	4.3 (6.2)	193	130.0 (34.3)	177	15.4 (8.6)
Father	43	83.7 (19.4)	41	3.2 (4.5)	81	134.2 (32.1)	73	16.5 (8.9)
Informant’s education								
Low	1	120	1	16	20	113.3 (33.4)	19	14.6 (8.8)
Middle	128	83.3 (26.4)	117	4.3 (6.7)	100	135.6 (33.2)	90	16.5 (8.4)
High	197	86.1 (22.0)	186	4.0 (5.6)	41	135.4 (32.0)	39	15.1 (8.6)
Total								
Baseline assessment	326	85.1 (23.9)	304	4.1 (6.0)	193	130.0 (34.3)	177	15.4 (8.6)
Six-month follow-up	165	88.1 (25.9)	156	4.3 (6.0)	–	–	–	–

Scores for the community sample include both mother or father reports. Scores for the clinical sample were based on mothers reports, except for the informant category, father scores are based on the same children; Means in the same column having the same superscript are significantly different at *p* < .05

#### Informant Differences in Clinical Sample

Both parents of 79 children completed the ECBI. Significant correlations were found between mother and father reports for the Intensity Scale (*r* (79) = .57, *p* < .001) and Problem Scale (*r*_*S*_ (73) = .49, *p* < .001). No significant effect of the informant’s gender was found for the total clinical sample, however, a paired sample *t*-test for the group with the mother and father reports (*n* = 79) revealed a significant difference (*t* (78) = 2.18, *p* = .03) for the Intensity Scale. Mothers reported a higher frequency of their child’s behavior problems than fathers (mothers, *M* = 142.1, *SD* = 31.3; fathers, *M* = 134.9, *SD* = 32.2). No significant differences were found on the Problem Scale (mothers, *M* = 16.7, *SD* = 8.3; fathers, *M* = 16.8, *SD* = 8.6).

#### Reproducibility in the Community Sample

Test-retest reliability was calculated for the 165 children in the community sample for whom the ECBI was completed at baseline and again 6 months later. Significant correlations between baseline and follow-up assessments were found for the Intensity Scale (*r* (165) = .84, *p* < .001) and Problem Scale (*r*_*S*_ (156) = .60, *p* < .001). Paired *t*-tests revealed a stable pattern of behavior over time for both scales (IS; *t* (164) = −.63, *p* = .53, PS; *t* (155) = −.16, *p* = .87). The reproducibility of the items and scale scores using weighted kappa and intraclass correlations are presented in Table 3. Kappa coefficients of the individual items indicated moderate to high reproducibility over 6 months. Weighted kappa coefficients ranged from 0.39 (item 21, *Steals*) to 0.76 (item 36, *Wets the bed*) for the ECBI Intensity Scale. The unweighted kappa for the ECBI Problem scale ranged from 0.25 (item 8, *Does not obey house rules on own*) to 0.56 (item 31, *Has short attention span*). Although some individual items had slightly lower kappa coefficients indicating moderate reproducibility, the intraclass correlations (ICC) between the baseline and follow-up assessments for the ECBI Intensity and Problem Scales were generally high (Table 3).

**Table 3:** Reproducibility of the item and total scale scores for the ECBI scales for the community sample

		Intensity scale (*n* = 165)	Problem scale (*n* = 160)
		Weighted Kappa	Unweighted Kappa
1.	Dawdles in getting dressed	0.656	0.393
2.	Dawdles or lingers at mealtime	0.581	0.500
3.	Has poor table manners	0.593	0.524
4.	Refuses to eat food presented	0.672	0.479
5.	Refuses to do chores when asked	0.485	0.306
6.	Slow in getting ready for bed	0.598	0.525
7.	Refuses to go to bed on time	0.474	0.407
8.	Does not obey house rules on own	0.493	0.249
9.	Refuses to obey until threatened with punishment	0.648	0.481
10.	Acts defiant when told to do something	0.538	0.375
11.	Argues with parents about rules	0.530	0.431
12.	Get angry when doesn’t get own way	0.584	0.452
13.	Has temper tantrums	0.654	0.465
14.	Sasses adults	0.570	0.357
15.	Whines	0.491	0.375
16.	Cries easily	0.711	0.458
17.	Yells or screams	0.702	0.506
18.	Hits parents	0.661	0.296
19.	Destroys toys and other objects	0.650	0.525
20.	Is careless with toys and other objects	0.557	0.348
21.	Steals	0.386	0.529
22.	Lies	0.514	0.376
23.	Teases or provokes other children	0.641	0.536
24.	Verbally fights with friends own age	0.577	0.341
25.	Verbally fights with sisters and brothers	0.659	0.445
26.	Physically fights with friends own age	0.532	0.341
27.	Physically fights with sisters and brothers	0.591	0.419
28.	Constantly seeks attention	0.666	0.459
29.	Interrupts	0.502	0.260
30.	Is easily distracted	0.661	0.411
31.	Has short attention span	0.673	0.561
32.	Fails to finish tasks or projects	0.710	0.391
33.	Has difficulty entertaining self alone	0.686	0.355
34.	Has difficulty concentrating on one thing	0.732	0.466
35.	Is overactive or restless	0.634	0.463
36.	Wets the bed	0.756	0.381
Intraclass correlation (ICC)		0.84	0.74

Kappa coefficients and Intraclass correlations for the community sample were calculated using baseline and follow-up scores

#### Convergent and Divergent Validity in the Clinical Sample

To examine the convergent and divergent validity of the ECBI scales in the clinical sample, correlations were calculated between the scores from the ECBI scales and the scores from the SDQ scales (see Table 4). The pattern of the correlation coefficients with regards to convergent validity were as hypothesized. The convergence between the ECBI Intensity Scale and the SDQ Conduct Problem and Hyperactivity/Impulsiveness scales ranged from *r*_*S*_ = .46 to .75. For the ECBI Problem Scales the convergence with these scales ranged from *r*_*S*_ = .36 to .62.

**Table 4:** Correlations between ECBI Intensity and Problem Scales and SDQ scales in the clinical sample

	Strengths and Difficulties Questionnaire (SDQ)							
	*n*	TOT	CON	HYP	CON+HYP	EMO	PEER	PRO
ECBI mother reports								
Intensity	192	0.67	0.65	0.63	0.75	0.26	0.13^ns^	−0.44
Problem	176	0.62	0.53	0.46	0.63	0.37	0.14^ns^	−0.19^ns^
ECBI father reports								
Intensity	79	0.54	0.57	0.48	0.62	0.19	0.09^ns^	−0.39
Problem	71	0.40	0.46	0.36	0.50	0.12^ns^	0.03^ns^	−0.10^ns^

*TOT* SDQ total difficulties scale, *CON* SDQ conduct problems scale, *HYP* SDQ hyperactivity/inattention scale, *CON+HYP* pooled SDQ conduct problems and hyperactivity/inattention scale, *EMO* SDQ emotional symptoms scale, *PEER* SDQ peer problems scale, *PRO* SDQ prosocial behavior scale

All correlations without a superscript were significant at *p* < .001; ^ns^ = no significant correlation

For all scales, correlations were lower between measures completed by fathers than those completed by mothers. Mothers were more likely to report similar behavior problems on the ECBI and SDQ than fathers. As expected, Table 4 shows higher correlations for the externalizing behavior SDQ scales compared to the SDQ Emotional Symptoms Scale (*r*_*S*_ = .12 to .37) and the SDQ Peer Problems Scale (*r*_*S*_ = .03 to .14). Also, the ECBI scales (and in particular the IS) were negatively correlated with the SDQ Prosocial Behavior Scale (*r*_*S*_ = −.10 to −.44).

#### Discriminative Validity in the Clinical Sample

Diagnostic information was available for 137 children (70 %). Fifty-one children (37.5 %) had no symptoms that met the criteria for a disruptive behavior disorder. Based on DSM-IV criteria, 32 children (23.4 %) were classified with significant attention deficit hyperactivity disorder symptoms (ADHD), nine children (6.6 %) were classified with significant oppositional defiant disorder symptoms (ODD), and two children (1.5 %) with conduct disorder symptoms (CD). Thirty-one children (22.6 %) had both significant ODD and ADHD symptoms, two children had significant ODD and CD symptoms, and two children had both significant ADHD and CD problems. In eight children (5.8 %) significant symptoms of all three disorders (ADHD, ODD & CD) were found.

To assess the ability of the ECBI Intensity and Problem Scale to differentiate between different behavioral disorders within the externalizing problems spectrum, mean scores for each diagnostic group were calculated (Weis et al. 2005). As a consequence of incomplete diagnostic data, children with no diagnostic information were excluded from these analyses. Children who met criteria for more than one DSM-IV disorder (ADHD, ODD & CD) were classified into the highest severity group. Severity ranges were assigned based on existing literature (Ross et al. 1998) with severity increasing from ADHD to ODD, and finally to CD as the most severe disorder. Mean scores for the ECBI Intensity and Problem Scales are presented in Table 5. One-way between-groups analyses of variance (ANOVA) revealed significant differences between diagnostic groups on the ECBI Intensity Scale *F*(3, 119) = 29.81, *p* < .001 and ECBI Problem Scale *F*(3, 119) = 16.67, *p* < .001. Post-hoc comparisons showed significant differences on both ECBI scales for children with no diagnosis and children with significant DSM-IV externalizing behavior symptoms. The ECBI Intensity Scale distinguished between three groups, based on the presence of symptoms: (1) children without significant externalizing symptoms, (2) children with significant ADHD symptoms, and (3) children with significant ODD and CD behavior symptoms. The ECBI Problem Scale was not able to differentiate between the different behavioral disorders within the externalizing problems spectrum, but it could differentiate between children with and without clinical symptoms of ADHD, ODD, or CD.

**Table 5:** Means and standard deviations of ECBI Intensity and Problem Scale by clinician assessed significant DSM-IV symptoms (*n* = 137)

	Clinician assessed symptoms			
	No diagnosis (*n* = 51)	ADHD (*n* = 32)	ODD (*n* = 39)	CD (*n* = 14)
	*M (SD)*	*M (SD)*	*M (SD)*	*M (SD)*
ECBI mother reports				
Intensity	111.40 (24.36)^a^	134.38 (23.64)^b^	157.40 (28.30)^c^	162.32 (24.71)^c^
Problem	10.72 (7.42)^a^	16.53 (7.43)^b^	20.43 (6.93)^b^	23.0 (5.35)^b^

Results in this table are mother reports from the clinical sample. Scores in the same row having an identical superscript are not significantly different at *p* < .05

## Discussion

The purpose of the current study was to investigate the psychometric properties of the ECBI in Dutch children. The dimensionality, internal consistency, test-retest reliability (reproducibility), convergent, divergent and discriminative validity were examined and our results provide evidence for good psychometric qualities of the ECBI in the Netherlands. This is in line with our hypotheses and the previous findings from other international studies.

Findings from this study confirm the one-dimensional structure of the ECBI Intensity and Problem Scales when measuring overall child disruptive behavior in a Dutch community and clinical population. These findings were supported by both classic psychometric tests (e.g. exploratory factor analyses, internal consistency) and modern psychometric tests (Rasch analysis, OPLM). Results confirm the use of the preferred one-factor scale in treatment outcome studies when compared to the three-factor structure. Due to the fact that these modern test analysis techniques are less dependent on sample characteristics, the generalizability of these results is high. These findings also support the use of the ECBI for screening and assessment purposes, because the ECBI Intensity and Problem Scales were able to discriminate between children with and without significant symptoms of ADHD, ODD or CD.

Good convergent and divergent validity of the ECBI Intensity and Problem Scales were found with the SDQ in the clinical sample. This is similar with results found by Axberg et al. (2008) in a Swedish community sample. Also, in other studies which examined the correlations of the ECBI Intensity Scale and Problem Scale with other scales for child behavior problems (e.g. Boggs et al. 1990; Funderburk et al. 2003). The strong correlations between the Intensity and Problems Scales (ranging from 0.62 to 0.75) found in the present study, the similar pattern of correlations found for the construct validity, and the similarity of the patterns over different informants (mothers and fathers), raise questions about the usefulness of keeping both ECBI scales separate. Given the parsimony criteria, it can be suggested to combine both scales into a single scale. In contrast with this suggestion, Eyberg (1992) stressed the importance of both ECBI scales which measure related but also include distinct dimensions of disruptive behavior in children. Eyberg (1992) suggested that parental perceptions are the underlying construct of the development of the separate scales. The ECBI Intensity and Problem Scales may be especially useful in regard to parental tolerance (McMahon and Frick 2007). Parents with a low Intensity score in conjunction with a high Problem Scale score may indicate high parenting stress or intolerance with the child’s behavior. On the other hand, parents with a high Intensity score and a low Problem score have a high tolerance level or are reluctant to acknowledge the behavior problems of their child. Although the ECBI scales can be useful with respect to parental perceptions, future research should study the added value of using both scales in treatment effectiveness research and screening proposes. Using additional measures to assess child behavior problems, such as observational measures in combination with a questionnaire assessing parental distress and perceptions, like the Parenting Stress Index (PSI; Abidin 1995) is recommended.

Findings regarding informant differences were contradictory to previous research (Colvin et al. 1999). In our clinical sample, mothers reported higher frequencies of disruptive behavior problems than fathers, for the same child. A possible reason for the tendency of mothers to report higher frequencies of child disruptive behavior is the mother’s role as primary caregiver. Due to the fact that mothers spend more time with their children, higher reported frequencies may be a consequence of more exposure to the problem behavior of the child (Biller 1993). Another possible reason for the discrepancies between the mother and father reports could be differences in the child’s behavior in the presence of the parents. It has been previously found that behavior problem children are more likely to comply when with their fathers (Campbell 2006; Patterson and Maccoby 1980). Fathers may, therefore, rate their children’s problem behavior as less frequent.

### Implications

These results suggest the possibility of weighing items when using them for screening purposes. If parents report a high frequency of a specific behavior on an item with a high weight (for example item 13, *has temper tantrums*), this child is likely the have a high total score on the ECBI Intensity Scale. Asking parents about the frequency of their child’s temper tantrums would be an easy way to identify young children at risk for severe disruptive behavior problems and then refer these families for preventive treatment. This is, however, a new direction with regards to the use of the ECBI scales, further research on this item weighting system is needed.

Considering the suggestions made by Axberg et al. (2008), minor changes are suggested in the Dutch ECBI version. For example, a checkbox for the sibling items (25 & 27), where a rater can indicate whether these questions are applicable to their child, would be useful. This would result in fewer missing items. Additionally, the low item statistics on item 36 *(wets the bed)* suggests that further explanation of this specific item would be helpful and a checkbox may again be useful. Then raters can indicate whether this question is applicable to the child, for example some children still wear a diaper during the night. These suggested changes might, however, affect the total ECBI Intensity and Problem scores, and therefore further consideration on the changes is required.

Finally, with regards to the normative data, the ECBI Intensity and Problem Scale means for the total community sample were significantly lower than the US norms found by Colvin and colleagues (1999) (IS; *t* (604) = 5.83, *p* < .001, PS; *t* (582) = 4.02, *p* < .001). This finding is similar to those found in other northern or western European studies which have explored ECBI norms (Axberg et al. 2008; Reedtz et al. 2008). Reconsideration of the ECBI Intensity and Problem scale cut-off points may also be helpful with regards to clinical assessment and treatment outcome studies.

### Strengths and Limitations

Our study has several strengths. Firstly, the inclusion of both a community and clinical sample provided information about different populations, which contributed to the generalizability of the study results within a Dutch population. Secondly, the multi-ethnic clinical sample was representative of the composition of other populations in other urban regions in western European countries. Thirdly, the use of modern test analysis techniques, which are less dependent on the specific characteristics of the samples, are an important strength with regards to the generalizability of the study results on the one-dimensional structure of the ECBI.

However, this present study has a number of limitations. First, in the community sample fewer children from ethnic minority groups were included and the response rate was partly unknown. The response rate was therefore small and the attrition rate (49.4 %) for the 6-month test-retest was high. Consequently, there is a lack of information about the generalizability of our findings, especially on the mean scores. Additional research on the psychometric properties and the mean scores in a multi-ethnic community sample with more focus on the response rate and the prevention of attrition is therefore recommended. Furthermore, in comparison with the clinical sample, the ECBI scales were not normally distributed in the community sample. However, non-normal distributions of scores are common in community samples because low answer categories (*never*) are chosen more frequently. As a consequence of the limited variation in the community sample and the relatively small sample sizes, we chose to combine data from both samples in the IRT analysis.

A final limitation is the way in which the clinical diagnoses were conducted by trained clinicians. Children in the clinical sample were from different child mental health centers. Although all clinicians used structured interviews according to DSM-IV criteria, no standardized procedure was used to assess the significant symptoms of ADHD, ODD and CD in the Dutch children. We have therefore chosen to use the term classifications rather than diagnoses. Nevertheless, results regarding the classifications should be interpreted with caution, as is common practice in Dutch clinical practice.

## Conclusion

The results of the current study provide evidence that the ECBI is a psychometrically sound measure for indicating disruptive behavior problems in children in the Netherlands. Data suggests that the ECBI Intensity and Problem Scales are internally consistent and appropriately correlated with another well-established questionnaire (the SDQ). The ECBI Intensity Scale is also able to differentiate between diagnostic groups within the externalizing behavior spectrum. Based on the evidence found for the one-dimensional structure of the ECBI, the original defined ECBI Intensity and Problem Scales are useful for screening and intervention research purposes in a Dutch population. The use of weighted items could also improve the use of the ECBI for screening purposes and clinical research, but further investigation on this new area is recommended.

## References

[CR1] Abidin RR (1995). Parenting stress index: Professional manual.

[CR2] Achenbach TM, Rescorla LA (2000). Manual for the ASEBA preschool forms and profiles.

[CR3] American Psychiatric Association (1994). Diagnostic and statistical manual of mental disorders.

[CR4] American Psychiatric Association (2013). Diagnostic and statistical manual of mental disorders.

[CR5] Aos S, Lieb R, Mayfield J, Miller M, Pennucci A (2004). Benefits and costs of prevention and early intervention programs for youth.

[CR6] Axberg U, Johansson Hanse J, Broberg AG (2008). Parents’ description of conduct problems in their children: a test of the Eyberg Child Behavior Inventory (ECBI) in a Swedish sample aged 3–10. Scandinavian Journal of Psychology.

[CR7] Biller HB (1993). Fathers and families: Paternal factors in child development.

[CR8] Boggs SR, Eyberg S, Reynolds LA (1990). Concurrent validity of the Eyberg Child Behavior Inventory. Journal of Clinical Child Psychology.

[CR9] Broidy LM, Nagin DS, Tremblay RE, Bates JE, Brame B, Dodge KA (2003). Developmental trajectories of childhood disruptive behaviors and adolescent delinquency: a six-site, cross-national study. Developmental Psychology.

[CR10] Burke JD, Waldman I, Lahey BB (2010). Predictive validity of childhood oppositional defiant disorder and conduct disorder: implications for the DSM-V. Journal of Abnormal Psychology.

[CR11] Burns GL, Patterson DR (1991). Factor structure of the Eyberg Child Behavior Inventory: unidimensional or multidimensional measure of disruptive behavior?. Journal of Clinical Child Psychology.

[CR12] Burns GL, Patterson DR (2000). Factor structure of the Eyberg Child Behavior Inventory: a parent rating scale of oppositional defiant behavior toward adults, inattentive behavior, and conduct problem behavior. Journal of Clinical Child Psychology.

[CR13] Butler AM (2011). Cross-racial measurement equivalence of the Eyberg Child Behavior Inventory factors among low-income young African American and Non-Latino White children. Assessment.

[CR14] Campbell, S. B. (2006). *Behavior problems in preschool children: Clinical and developmental issues*. New York: Guilford Press.

[CR15] Colvin, A., Eyberg, S. M., & Adams, C. D. (1999). *Restandardization of the Eyberg Child Behavior Inventory*. Gainesville, FL: University of Florida, Child Study Laboratory.

[CR16] Comer JS, Chow C, Chan PT, Cooper-Vince C, Wilson LA (2013). Psychosocial treatment efficacy for disruptive behavior problems in very young children: a meta-analytic examination. Journal of the American Academy of Child and Adolescent Psychiatry.

[CR17] Eisenstadt TH, Eyberg S, McNeil C, Newcomb K, Funderburk B (1993). Parent–child interaction therapy with behavior problem children: relative effectiveness of two stages and overall treatment outcome. Journal of Clinical Child Psychology.

[CR18] Eyberg SM, VandeCreek L, Knapp S, Jackson TL (1992). Parent and teacher behavior inventories for the assessment of conduct problem behaviors in children. Innovations in clinical practice: A source book.

[CR19] Eyberg SM, Pincus DB (1999). Eyberg child behavior inventory and Sutter-Eyberg behavior inventory-revised: Professional manual.

[CR20] Eyberg SM, Robinson EA (1983). Conduct problem behavior: standardization of a behavioral rating. Journal of Clinical Child Psychology.

[CR21] Eyberg SM, Nelson MM, Boggs SR (2008). Evidence-based psychosocial treatments for children and adolescents with disruptive behavior. Journal of Clinical Child and Adolescent Psychology.

[CR22] Fabrigar LR, Wegener DT, MacCallum RC, Strahan EJ (1999). Evaluating the use of exploratory factor analysis in psychological research. Psychological Methods.

[CR23] Fleiss, J. L., & Cohen, J. (1973). The equivalence of weighted kappa and the intraclass correlation coefficient as measures of reliability. *Educational and Psychological Measurement*, *33*, 613-619.

[CR24] Funderburk BW, Eyberg SM, Rich BA, Behar L (2003). Further psychometric evaluation of the Eyberg and Behar rating scales for parents and teachers of preschoolers. Early Education and Development.

[CR25] Goodman R (1997). The strengths and difficulties questionnaire: a research note. Journal of Child Psychology and Psychiatry.

[CR26] Gross D, Fogg L, Young M, Ridge A, Cowell J, Sivan A (2007). Reliability and validity of the Eyberg Child Behavior Inventory with African-American and Latino parents of young children. Research in Nursing and Health.

[CR27] Heckman JJ (2006). Skill formation and the economics of investing in disadvantaged children. Science.

[CR28] Horn JL (1965). A rationale and test for the number of factors in factor analysis. Psychometrika.

[CR29] Lee S, Aos S, Drake E, Pennucci A, Miller U, Anderson L (2012). Return on investment: Evidence-based options to improve statewide outcomes.

[CR30] McMahon RJ, Frick PJ, Mash EJ, Barkley RA (2007). Conduct and oppositional disorder. Assessment of childhood disorders.

[CR31] Michelson D, Davenport C, Dretzke J, Barlow J, Day C (2013). Do evidence-based interventions work when tested in the “real world?” a systematic review and meta-analysis of parent management training for the treatment of child disruptive behavior. Clinical Child and Family Psychology Review.

[CR32] Nixon RD, Sweeney L, Erickson DB, Touyz SW (2004). Parent–child interaction therapy: one- and two-year follow-up of standard and abbreviated treatments for oppositional preschoolers. Journal of Abnormal Child Psychology.

[CR33] Nolan EE, Gadow KD, Sprafkin J (2001). Teacher reports of DSM-IV ADHD, ODD, and CD symptoms in schoolchildren. Journal of the American Academy of Child and Adolescent Psychiatry.

[CR34] Patterson, G. R., & Maccoby, E. E. (1980). Mothers: the unacknowledged victims. *Monographs of the Society for Research in Child Development*, *45, * 1–64.

[CR35] Rasch G (1960). Probabilistic models for some intelligence and attainment tests.

[CR36] Reedtz C, Bertelsen B, Lurie J, Handegard BH, Clifford G, Morch WT (2008). Eyberg Child Behavior Inventory (ECBI): Norwegian norms to identify conduct problems in children. Scandinavian Journal of Psychology.

[CR37] Ross CN, Blanc HM, McNeil CB, Eyberg SM, Hembree-Kigin TL (1998). Parenting stress in mothers of young children with oppositional defiant disorder and other severe behavior problems. Child Study Journal.

[CR38] Scott S, Knapp M, Henderson J, Maughan B (2001). Financial cost of social exclusion: follow up study of antisocial children into adulthood. BMJ.

[CR39] Sivan AB, Ridge A, Gross D, Richardson R, Cowell J (2008). Analysis of two measures of child behavior problems by African American, Latino, and non-Hispanic Caucasian parents of young children: a focus group study. Journal of Pediatric Nursing.

[CR40] Statistics Netherlands. (2013). Overview definition for people with a foreign background. http://www.cbs.nl/enGB/menu/themas/dossiers/allochtonen/methoden/begrippen/default.htm?Languageswitch=on&ConceptID=37. Accessed 25 Nov 2013.

[CR41] Statistics Netherlands. (2014). Overview definition for educational level. http://www.cbs.nl/NR/rdonlyres/7C94DE33-621C-4355-928A8B90F9F5D777/0/2006soiniveauindeling201213.pdf. Accessed 23 May 2014.

[CR42] Verhelst ND, Glas CA, Fischer G, Molenaar I (1995). The one parameter logistic model. Rasch models, foundations, recent developments and applications.

[CR43] Verhelst ND, Glas CA, Verstalen H (2005). OPLM: One parameter logistic model: computer program and manual.

[CR44] Weis R, Lovejoy MC, Lundahl B (2005). Factor structure and discriminative validity of the Eyberg Child Behavior Inventory with young children. Journal of Psychopathology and Behavioral Assessment.

